# Supplementary material for: Predicting how and when hidden neurons skew measured synaptic interactions
Source: PLoS Comput Biol. 2018 Oct 22;14(10):e1006490. doi: 10.1371/journal.pcbi.1006490 (PMC6219819; doi:10.1371/journal.pcbi.1006490)
Supplement: S1 Table — (PDF) [file pcbi.1006490.s004.pdf]

Table S1. Network activity simulation parameter values.

Network connectivity parameters	See Table 1.
Alpha function decay time $\tau \equiv 1/\alpha$	10
Time bin width Δt	0.01τ
Transient time window	5τ
Simulation stopping time	4000τ + transient
