# Supplementary material for: Predicting how and when hidden neurons skew measured synaptic interactions
Source: PLoS Comput Biol. 2018 Oct 22;14(10):e1006490. doi: 10.1371/journal.pcbi.1006490 (PMC6219819; doi:10.1371/journal.pcbi.1006490)
Supplement: S1 Text — (PDF) [file pcbi.1006490.s005.pdf]

Supporting Information for “Predicting How and When Hidden Neurons Skew Measured Synaptic Interactions”

Braden A. W. Brinkman[1*,†], Fred Rieke[1,2], Eric Shea-Brown[1,2,3], Michael A. Buice[5,3]

1 Department of Neurobiology and Behavior, Stony Brook University, Stony Brook, NY, 11794, USA
2 Department of Physiology and Biophysics, University of Washington, Seattle, WA, 98195, USA
3 Graduate Program in Neuroscience, University of Washington, Seattle, WA, 98195, USA
4 Department of Applied Mathematics, University of Washington, Seattle, WA, 98195, USA
5 Allen Institute for Brain Science, Seattle, WA, 98109, USA
† Previously affiliated at 4, 2.

* bradenb@stonybrook.edu

Mapping a leaky integrate-and-fire network with stochastic spiking to the nonlinear Hawkes model

As claimed in the Methods, we now show explicitly how to map a current-based leaky-integrate and fire network model with stochastic spiking rules on to the nonlinear Hawkes model we use in this work. Suppose each neuron's membrane potential obeys the differential equation

$$\tau_m \frac{dV_i}{dt} = -(V_i - \mathcal{E}_L) + \mathcal{E}_i^{\rm syn}(t) + \mathcal{E}_i^{\rm ext}(t), \tag{S1}$$

where τ_m is the membrane time constant of the neuron, $\mathcal{E}_L$ is its reversal potential, $\mathcal{E}_i^{\rm ext}(t)$ is an external current input (converted to a voltage by dividing by the membrane resistance), and

$$\mathcal{E}_i^{\rm syn}(t) = \sum_j \int_{-\infty}^{t} dt'~\tilde{J}_{ij}(t-t')\dot{n}_j(t')$$

are the synaptic currents flowing into neuron i from presynaptic neurons j, where $\dot{n}_j(t')$ is the spike train from presynaptic neuron j at time t' and $\tilde{J}_{ij}(t-t')$ is a spike filter. For notational convenience we also include the self-history coupling $\tilde{J}_{i,i}(t-t')$ in this term, though it has a physiologically different origin, representing refractory effects that reset a neuron's voltage after it spikes, rather than having a hard reset. Similarly, rather than having a hard firing threshold, we assume that neurons spike stochastically with an instantaneous rate

$$\lambda_i(t) = \lambda_0 \phi\left(\frac{V_i(t) - \mathcal{E}_{\rm th}}{\mathcal{E}_{\rm s}}\right),$$

where λ_0 sets a baseline firing rate, $\phi(\cdot) \geq 0$ is a nonlinear function of the membrane voltage, $\mathcal{E}_{\rm th}$ is a “soft” threshold value, $\mathcal{E}_{\rm s}$ sets the steepness of the nonlinearity, and $V_i(t)$ is the membrane voltage given in Eq. (S1). We term this the instantaneous firing rate because it is equal to the the trial-averaged spike trains $\dot{n}_i(t)$, conditioned on the

inputs to the neuron. The value $\mathcal{E}_{\rm th}$ is a soft-threshold because while it is likely the neuron will fire when $V_i(t)$ reaches $\mathcal{E}_{\rm th}$, it is possible the neuron will fire at higher or lower voltage. In this work we assume that the probability that the number of spikes neuron i fires in a small time window Δt around time t, given its input history, is

$$\dot{n}_i(t)\Delta t \sim \text{Poiss}\left[\lambda_i(t)\Delta t\right];$$

however, we could have chosen many other point processes with instantaneous rates $\lambda_i(t)$. Note that while spiking is stochastic, a neuron is guaranteed to fire at times when its instantaneous rate $\lambda_i(t)$ diverges, so there is some sense of deterministic output retained.

We can formally solve the membrane equation (S1), giving

$$V_i(t) = \mathcal{E}_L + \int_{-\infty}^{t} dt'' \ \frac{e^{-(t-t'')/\tau_m}}{\tau_m} \left[\mathcal{E}_{\rm ext}(t'') + \sum_j \int_{-\infty}^{t''} dt' \ \tilde{J}_{ij}(t''-t')\dot{n}_j(t')\right].$$

We now define

$$\mu_i = \frac{\mathcal{E}_L - \mathcal{E}_{\rm th}}{\mathcal{E}_{\rm s}},$$

$$\mu_i^{\rm ext}(t) = \frac{\int_{-\infty}^{t} dt'' \ \frac{e^{-(t-t'')/\tau_m}}{\tau_m}\mathcal{E}_i^{\rm ext}(t'')}{\mathcal{E}_{\rm s}},$$

and

$$J_{ij}(t-t') = \frac{\frac{e^{-(t-t')/\tau_m}}{\tau_m}\int_0^{t-t'} dy \ \frac{e^{y/\tau_m}}{\tau_m}\tilde{J}_{ij}(y)}{\mathcal{E}_{\rm s}};$$

we arrive at this last definition by changing integration order

$$\begin{aligned}&\int_{-\infty}^{t} dt'' \ \frac{e^{-(t-t'')/\tau_m}}{\tau_m}\int_{-\infty}^{t''} dt' \ \tilde{J}_{ij}(t''-t')\dot{n}_j(t')\\ =&\int_{-\infty}^{t} dt' \ \left\{\int_{t'}^{t} dt'' \ \frac{e^{-(t-t'')/\tau_m}}{\tau_m}\tilde{J}_{ij}(t''-t')\right\}\dot{n}_j(t')\end{aligned}$$

and then changing variables to $y = t - t''$. With these definitions,

$$\lambda_i(t) = \lambda_0\phi\left(\mu_i + \mu_i^{\rm ext}(t) + \sum_j \int_{-\infty}^{t} dt' \ J_{ij}(t-t')\dot{n}_j(t')\right);$$

i.e., we have shown the the soft-threshold leaky integrate-and-fire model is equivalent to a nonlinear Hawkes model, Eq. (5). Because the argument of the rate is now expressed entirely in terms of the spiking of the neurons, and not the membrane voltage, we need only simulate the spiking activity of the network; i.e., we do not need to keep track of the membrane voltages and can simply use Eq. (5).

Lastly, we note that membrane potential dynamics are more appropriately described by changes to a neuron's membrane conductance, rather than current inputs [1]. If we insert conductance-based synaptic inputs, such as

$$\mathcal{E}_i^{\rm syn}(t) = -(V_i(t) - \mathcal{E}_i^S)\sum_j \int_{-\infty}^{t} dt' \ \tilde{J}_{ij}^{\rm cond}(t-t')\dot{n}_j(t'),$$

into Eq. (S1), the voltage equation is still formally solvable, but the rates $\lambda_i(t)$ will no longer be of the form of Eq. (5), except in approximate limits or if special conditions are met [2]. We leave a more detailed investigation of conductance-based models—including those with nonlinear voltage dependence—for future work.

Complete derivation of the contribution of self-cycles to nodes in Fig. 2

In the Methods section of the full text, we heuristically argued that loops from a neuron back to itself in the series expansion of $\hat{\Gamma}_{h,h'}(\omega) = [\mathbb{I} - \hat{\mathbf{V}}(\omega)]^{-1}_{h,h'}\gamma_{h'}$ could be explicitly summed into a factor $1/(1 - \gamma_h \hat{J}_{h,h}(\omega))$ contributed by each node h. This factor can be derived directly; we do so here.

Let us decompose the matrix $\hat{\mathbf{V}}(\omega)$ in a diagonal and off-diagonal piece, $\hat{\mathbf{V}}(\omega) = \hat{\mathbf{V}}_{\text{diag}}(\omega) + \hat{\mathbf{V}}_{\text{off}}(\omega)$. Then,

$$\begin{aligned}\left[\mathbb{I} - \hat{\mathbf{V}}(\omega)\right]^{-1} &= \left[\mathbb{I} - \hat{\mathbf{V}}_{\text{diag}}(\omega) - \hat{\mathbf{V}}_{\text{off}}(\omega)\right]^{-1} \\ &= \left[\left(\mathbb{I} - \hat{\mathbf{V}}_{\text{diag}}(\omega)\right)\left(\mathbb{I} - \left(\mathbb{I} - \hat{\mathbf{V}}_{\text{diag}}(\omega)\right)^{-1}\hat{\mathbf{V}}_{\text{off}}(\omega)\right)\right]^{-1} \\ &= \left[\mathbb{I} - \left(\mathbb{I} - \hat{\mathbf{V}}_{\text{diag}}(\omega)\right)^{-1}\hat{\mathbf{V}}_{\text{off}}(\omega)\right]^{-1}\left[\mathbb{I} - \hat{\mathbf{V}}_{\text{diag}}(\omega)\right]^{-1}\end{aligned}$$

We assumed that $\mathbb{I} - \hat{\mathbf{V}}_{\text{diag}}(\omega)$ is invertible, which requires that there is no element for which $1 - \gamma_h \hat{J}_{h,h}(\omega) = 0$ for all ω. Assuming this is the case, the inverse of the matrix is trivial to calculate, as it is diagonal:

$$\left[\mathbb{I} - \hat{\mathbf{V}}_{\text{diag}}(\omega)\right]^{-1}_{h,h'} = \frac{1}{1 - \gamma_h J_{h,h}(\omega)}\delta_{h,h'}.$$

The matrix $\left[\mathbb{I} - \left(\mathbb{I} - \hat{\mathbf{V}}_{\text{diag}}(\omega)\right)^{-1}\hat{\mathbf{V}}_{\text{off}}(\omega)\right]^{-1}$ can be expressed as a series, as before:

$$\begin{aligned}&\left[\mathbb{I} - \left(\mathbb{I} - \hat{\mathbf{V}}_{\text{diag}}(\omega)\right)^{-1}\hat{\mathbf{V}}_{\text{off}}(\omega)\right]^{-1}_{h,h''} \\ =&\sum_{\ell=0}\left[\left[\left(\mathbb{I} - \hat{\mathbf{V}}_{\text{diag}}(\omega)\right)^{-1}\hat{\mathbf{V}}_{\text{off}}(\omega)\right]^{\ell}\right]_{h,h''} \\ =&\sum_{\ell=0}\sum_{h_1,\ldots,h_\ell}\left[\left(\mathbb{I} - \hat{\mathbf{V}}_{\text{diag}}(\omega)\right)^{-1}\hat{\mathbf{V}}_{\text{off}}(\omega)\right]_{h,h_1}\cdots\left[\left(\mathbb{I} - \hat{\mathbf{V}}_{\text{diag}}(\omega)\right)^{-1}\hat{\mathbf{V}}_{\text{off}}(\omega)\right]_{h_\ell,h''} \\ =&\sum_{\ell=0}\sum_{h_1,\ldots,h_\ell;h_i\neq h_{i+1}}\frac{\gamma_h}{1 - \gamma_h \hat{J}_{h,h}(\omega)}\hat{J}_{h,h_1}(\omega)\cdots\frac{\gamma_{h_\ell}}{1 - \gamma_{h_\ell}\hat{J}_{h_\ell,h_\ell}(\omega)}J_{h_\ell,h''}(\omega)\end{aligned}$$

Hence, inserting the contribution from the factor $\left[\mathbb{I} - \hat{\mathbf{V}}_{\text{diag}}(\omega)\right]^{-1}$ that we pulled out, and the factor $\gamma_{h'}$ that left-multiplies $\left[\mathbb{I} - \hat{\mathbf{V}}(\omega)\right]^{-1}$ to give $\hat{\Gamma}_{h,h'}(\omega)$, we have

$$\hat{\Gamma}_{h,h'}(\omega) = \sum_{\ell=0}\sum_{h_1,\ldots,h_\ell;h_i\neq h_{i+1}}\frac{\gamma_h \hat{J}_{h,h_1}(\omega)}{1 - \gamma_h \hat{J}_{h,h}(\omega)}\cdots\frac{\gamma_{h_\ell}\hat{J}_{h_\ell,h'}(\omega)}{1 - \gamma_{h_\ell}\hat{J}_{h_\ell,h_\ell}(\omega)}\frac{\gamma_{h'}}{1 - \gamma_{h'}\hat{J}_{h,h'}(\omega)}$$

This is the same as our previous expression, with $\gamma_h \to \gamma_h/(1 - \gamma_h \hat{J}_{h,h}(\omega))$ and restricting the sum over hidden units such that self-loops are removed ($h_i \neq h_{i+1}$), proving the result described informally above. We note again that this puts restrictions on the allowed size of self-interactions, as the zeros of $1 - \gamma_h \hat{J}_{h,h}(\omega)$ must be in the upper-half plane of the complex ω plane in order for the filters to be causal and physically meaningful (given our Fourier sign-convention $\hat{f}(\omega) = \int_{-\infty}^{\infty} dt\; e^{-i\omega t} f(t)$).

The complete expression for the correction term $\sum_{h,h'} \hat{J}_{r,h}(\omega)\hat{\Gamma}_{h,h'}(\omega)\hat{J}_{h',r'}(\omega)$ is thus

$$\sum_{h,h'} \hat{J}_{r,h}(\omega)\hat{\Gamma}_{h,h'}(\omega)\hat{J}_{h',r'}(\omega) =$$

$$\sum_{\ell=0} \sum_{h,h_1,\ldots,h_\ell,h';h_i\neq h_{i+1}} \hat{J}_{r,h}(\omega)\frac{\gamma_h \hat{J}_{h,h_1}(\omega)}{1-\gamma_h \hat{J}_{h,h}(\omega)} \cdots \frac{\gamma_{h_\ell} \hat{J}_{h_\ell,h'}(\omega)}{1-\gamma_{h_\ell} \hat{J}_{h_\ell,h_\ell}(\omega)} \frac{\gamma_{h'}}{1-\gamma_{h'} \hat{J}_{h,h'}(\omega)} \hat{J}_{h',r'}(\omega).$$

This is the exact mathematical expression underlying the graphical rules given in Fig. 2.

Second order nonlinear response function

Higher order terms in the series expansion represent nonlinear response functions. We do not focus on these terms in this work, assuming instead that we can truncate this series expansion at linear order. We will, however, estimate the error incurred by this truncation by calculating the second order response function, which we label $\Gamma^{(2)}_{h,h_1,h_2}(t,t_1,t_2)$. Rather than differentiate our formal solution for the linear response, we differentiate the implicit form, yielding an integral equation

$$\begin{aligned}\Gamma^{(2)}_{h,h_1,h_2}(t,t_1,t_2) &\equiv \left.\frac{\delta^2 \mathbb{E}[\dot{n}_h | \{I_h(t)\}]}{\delta I_{h_2}(t_2)\delta I_{h_1}(t_1)}\right|_{I_h=0} \\ &= \gamma^{(2)}_h \left[\delta_{h,h_2}\delta(t-t_2) + \sum_{h'} J_{h,h'} * \Gamma_{h',h_2}\right]\left[\delta_{h-h_1}\delta(t-t_1) + \sum_{h'} J_{h,h'} * \Gamma_{h',h_1}\right] \\ &\quad + \gamma_h \left[\sum_{h'} \int_{-\infty}^{\infty} dt' \; J_{h,h'}(t-t')\Gamma^{(2)}_{h,h_1,h_2}(t',t_1,t_2)\right]\end{aligned}$$

where we have defined

$$\gamma^{(2)}_h \equiv \lambda_0 \phi'' \left(\mu_h + \sum_{h'} \mathcal{J}_{h,h'}\nu_{h'}\right).$$

γ_h without the superscript is the gain defined previously, $\gamma_h = \lambda_0\phi'(\mu_h + \sum_{h'} \mathcal{J}_{h,h'}\nu_{h'})$. Rearranging,

$$\begin{aligned}&\int dt' \sum_{h'} \left[\delta_{h,h'}\delta(t-t') - \gamma_h J_{h,h'}(t-t')\right]\Gamma^{(2)}_{h',h_1,h_2}(t',t_1,t_2) \\ &\qquad = \gamma^{(2)}_h \left[\delta_{h-h_2}\delta(t-t_2) + \sum_{h'} J_{h,h'} * \Gamma_{h',h_2}\right]\left[\delta_{h-h_1}\delta(t-t_1) + \sum_{h'} J_{h,h'} * \Gamma_{h',h_1}\right].\end{aligned}$$

Inverting the operator on the left hand side yields the input linear response function (when introducing the factor of $1 = \gamma_{h'}/\gamma_{h'}$ on the right hand side), giving the solution

$$\begin{aligned}\Gamma^{(2)}_{h,h_1,h_2}(t,t_1,t_2) = \int_{-\infty}^{\infty} dt' \sum_{h'} \Gamma_{h,h'}(t-t')\frac{\gamma^{(2)}_{h'}}{\gamma_{h'}} &\left[\delta_{h',h_2}\delta(t'-t_2) + \sum_{h''}\int_{-\infty}^{\infty} dt'' \; J_{h',h''}(t'-t'')\Gamma_{h'',h_2}(t''-t_2)\right] \\ \times &\left[\delta_{h',h_1}\delta(t'-t_1) + \sum_{h''}\int_{-\infty}^{\infty} dt'' \; J_{h',h''}(t'-t'')\Gamma_{h'',h_1}(t''-t_1)\right]\end{aligned}$$

Because $\Gamma_{h,h'}(t-t')$ is proportional to γ_h, the second order nonlinear response function is proportional to $\gamma_h^{(2)}$. For an exponential nonlinearity, $\gamma_h^{(2)} = \gamma_h = \nu_h$, and the second order response function is of the same order of the linear response (but the overall contribution to network statistics is not of the same order; see below). For a rectified linear nonlinearity (as in Figs. 3 and 4), $\gamma_h^{(2)} = 0$ and the second-order response vanishes.

The effective quadratic interaction from two recorded neurons r_1' and r_2' to neuron r is thus

$$\int dt_1' dt_2' \sum_{r_1', r_2'} J^{(2)}_{r,r_1',r_2'}(t, t_1', t_2') \dot{n}_{r_1'}(t_1') \dot{n}_{r_2'}(t_2'),$$

where we have defined the quadratic spike interaction $J^{(2)}_{r,r_1',r_2'}(t, t_1', t_2')$ to be

$$J^{(2)}_{r,r_1',r_2'}(t, t_1', t_2') = \int dt' dt_1 dt_2 \sum_{h,h_1,h_2} J_{r,h}(t-t') \Gamma^{(2)}_{h,h_1,h_2}(t', t_1, t_2) J_{h_1,r_1'}(t_1-t_1') J_{h_2,r_2'}(t_2-t_2') \tag{S2}$$

Estimating the error from neglecting higher order spike filtering (exponential nonlinearity)

In the main text we calculate corrections to the baseline and linear spike filters, neglecting higher-order spike filtering and fluctuations around the mean input to the recorded neurons. In the methods we validated this result numerically; here we derive an analytic estimate of the order of the error we incur by neglecting these terms. We will do so within mean field theory (meaning the noise fluctuations contribute zero error as they do not contribute to the mean field approximation). Specifically, we will assume that the quadratic spike filtering term is small, and calculate the corresponding correction to our mean field approximation of the firing rates when this term is completely neglected. If we take as our approximation of the recorded neuron firing instantaneous firing rates

$$\lambda_r(t) \approx \lambda_0 \exp\Bigg(\mu_r^{\rm eff} + \sum_{r_1} \int dt_1 J^{\rm eff}_{r,r_1}(t-t_1) \dot{n}_{r_1}(t_1) + b \sum_{r_1,r_2} \int dt_1 dt_2 \; J^{(2)}_{r,r_1,r_2}(t, t_1, t_2) \dot{n}_{r_1}(t_1) \dot{n}_{r_2}(t_2) \Bigg),$$

then the mean field approximation of the firing rates is

$$\langle \dot{n}_r \rangle \approx \lambda_0 \exp\left(\mu_r^{\rm eff} + \sum_{r_1} \mathcal{J}^{\rm eff}_{r,r'} \langle \dot{n}_{r_1} \rangle + b \sum_{r_1,r_2} \mathcal{J}^{(2)}_{r,r_1,r_2} \langle \dot{n}_{r_1} \rangle \langle \dot{n}_{r_2} \rangle \right),$$

where we have used the fact that the average firing rates are independent of time, and replaced $J^{\rm eff}_{r,r'}(t-t_1)$ and $J^{(2)}_{r,r_1,r_2}(t, t_1, t_2)$ with their time integrals, denoted by $\mathcal{J}^{\rm eff}_{r,r'}$ and $\mathcal{J}^{(2)}_{r,r_1,r_2}$. The parameter b is just a book-keeping parameter.

To calculate the lowest order correction to the linear filtering approximation ($b \to 0$), we write $\langle \dot{n}_r \rangle = \nu_r^{\rm sub} + b\tilde{\nu}_r$, treating b formally as a small parameter. The linear firing rate $\nu_r^{\rm sub}$ is given by

$$\nu_r^{\rm sub} = \lambda_0 \exp\left(\mu_r^{\rm eff} + \sum_{r'} \mathcal{J}^{\rm eff}_{r,r'} \nu^{\rm sub}_{r'} \right).$$

For the quadratically-modified firing rates, keeping terms only to linear order in b,

$$\begin{aligned}\nu_r^{\mathrm{sub}} + b\tilde{\nu}_r &\approx \lambda_0 \exp\left(\mu_r^{\mathrm{eff}} + \sum_{r_1} \mathcal{J}^{\mathrm{eff}}_{r,r'}\nu^{\mathrm{sub}}_{r'} + b\sum_{r'} \mathcal{J}^{\mathrm{eff}}_{r,r'}\tilde{\nu}_{r'} + b\sum_{r_1,r_2} \mathcal{J}^{(2)}_{r,r_1,r_2}\nu^{\mathrm{sub}}_{r_1}\nu^{\mathrm{sub}}_{r_2}\right)\\ &= \nu_r^{\mathrm{sub}} \exp\left(b\sum_{r'} \mathcal{J}^{\mathrm{eff}}_{r,r'}\tilde{\nu}_{r'} + b\sum_{r_1,r_2} \mathcal{J}^{(2)}_{r,r_1,r_2}\nu^{\mathrm{sub}}_{r_1}\nu^{\mathrm{sub}}_{r_2}\right)\\ &\approx \nu_r^{\mathrm{sub}}\left\{1 + b\sum_{r'} \mathcal{J}^{\mathrm{eff}}_{r,r'}\tilde{\nu}_{r'} + b\sum_{r_1,r_2} \mathcal{J}^{(2)}_{r,r_1,r_2}\nu^{\mathrm{sub}}_{r_1}\nu^{\mathrm{sub}}_{r_2}\right\}.\end{aligned}$$

Collecting on b and rearranging,

$$\sum_{r'} \left[\delta_{r,r'} - \nu_r^{\mathrm{sub}}\mathcal{J}^{\mathrm{eff}}_{r,r'}\right]\tilde{\nu}_{r'} = \nu_r^{\mathrm{sub}} \sum_{r_1,r_2} \mathcal{J}^{(2)}_{r,r_1,r_2}\nu^{\mathrm{sub}}_{r_1}\nu^{\mathrm{sub}}_{r_2}.$$

Because $\nu_r^{\mathrm{sub}} \propto \exp(\mu_r^{\mathrm{eff}}) \propto \exp(\mu_r) = \epsilon_r$, the expansion parameters we have been using, the lowest order approximation for $\tilde{\nu}_r$ is

$$\tilde{\nu}_r \approx \nu_r^{\mathrm{sub}} \sum_{r_1,r_2} \mathcal{J}^{(2)}_{r,r_1,r_2}\nu^{\mathrm{sub}}_{r_1}\nu^{\mathrm{sub}}_{r_2}.$$

To evaluate the coefficient $\mathcal{J}^{(2)}_{r,r_1,r_2}$, we may use the fact $\gamma_h = \nu_h$ and to leading order $\nu_h \sim \lambda_0\epsilon$ and $\Gamma^{(2)}_{h,h_1,h_2}(t,t_1,t_2) \approx \lambda_0\epsilon\delta_{h,h_1}\delta_{h,h_2}\delta(t-t_1)\delta(t-t_2)$, giving

$$J^{(2)}_{r,r_1,r_2}(t,t_1,t_2) \approx \lambda_0\epsilon \int dt' \sum_h J_{r,h}(t-t')J_{h,r_1'}(t_1-t')J_{h,r_2'}(t_2-t')$$

and hence

$$\mathcal{J}^{(2)}_{r,r_1,r_2} \equiv \int dt_1 dt_2\; J^{(2)}_{r,r_1,_2}(t,t_1,t_2) \approx \lambda_0\epsilon \sum_h \mathcal{J}_{r,h}\mathcal{J}_{h,r_1}\mathcal{J}_{h,r_2}.$$

To lowest order the error term $\tilde{\nu}_r$ is

$$\tilde{\nu}_r = (\lambda_0\epsilon)^4 \sum_{h,r_1,r_2} \mathcal{J}_{r,h}\mathcal{J}_{h,r_1}\mathcal{J}_{h,r_2}.$$

For $\mathcal{J}_{i,j}$ *i.i.d.*, the population average should converge to the expected value, which is zero because the $\mathcal{J}_{i,j}$ have mean zero. We can calculate the root-mean-squared-error (RMSE) by looking at the variance:

$$\begin{aligned}\mathrm{var}(\tilde{\nu}_r) = \mathrm{var}\left(\sum_{h,r_1,r_2} J_{r,h}J_{h,r_1}J_{h,r_2}\right) &= \overline{\left(\sum_{h,r_1,r_2} \mathcal{J}_{r,h}\mathcal{J}_{h,r_1}\mathcal{J}_{h,r_2}\right)^2}\\ &= \sum_{h,r_1,r_2} \overline{\mathcal{J}^2_{r,h}}\;\overline{\mathcal{J}^2_{h,r_1}\mathcal{J}^2_{h,r_2}}\end{aligned}$$

In principle, we should take care to separate out the $r_1 \neq r_2$ and $r_1 = r_2$ terms from the sum, as the latter will contribute a factor $\overline{\mathcal{J}^4_{h,r_1}}$, which we have not specified yet (though one could calculate for specific choices, such as the normal distribution that we use for most of our numerical analyses). However, both $\overline{\mathcal{J}^2_{h,r}}^2$ and $\overline{\mathcal{J}^4_{h,r_1}}$ will scale as $(J_0^2/(pN)^{2a})^2$, so we will neglect constant factors and simply use this scaling to arrive at the result

$$\mathrm{var}(\tilde{\nu}_r) \sim (\lambda_0\epsilon)^8 N^2_{\mathrm{rec}} N_{\mathrm{hid}} \frac{J_0^6}{(pN)^{6a}}.$$

If we take $N \to \infty$ with $N_{\rm rec} = fN$ and $N_{\rm hid} = (1-f)N$ for f fixed, the RMSE scales as

$$\tilde{\nu}_{\rm RMSE} \sim (\lambda_0 \epsilon)^4 f \sqrt{1-f} \frac{J_0^3}{(pN)^{3a-3/2}}.$$

For $a = 1$ (weak coupling), the error falls off quite quickly as $N^{3/2}$, while it is independent of N for $a = 1/2$ (strong coupling). However, the error does still scale with the fraction of observed neurons, as $f\sqrt{1-f}$. This demonstrates that the typical error that arises from neglecting the nonlinear filtering is small both when most neurons have been observed ($f \lesssim 1$) and when very few neurons have been observed ($f \gtrsim 0$). While it may at first seem surprising that the error is small when very few neurons have been observed, the result does make intuitive sense: when a very small fraction of the network is observed, we can treat the unobserved portion of the network as a "reservoir" or "bath." Feedback from the observed neurons into the reservoir has a comparatively small effect, so we can get away with neglecting feedback between the observed and unobserved partitions of the network. However, when the number of observed neurons is comparable to the number of unobserved neurons, neither can be treated as a reservoir, and feedback between the two partitions is substantial. Neglecting the higher order spike filter terms may be inaccurate in this case.

Tree-level calculation of the effective noise correlations

In our approximation of the model for the recorded neurons, we also neglected fluctuations from the mean input around the hidden neuron input. We should therefore check how strong this noise is. At the level of a mean-field approximation we may neglect it, so we will need to go to a tree-level approximation to calculate it. (The means and response functions are not modified at tree-level.)

The noise is defined by

$$\xi_r(t) = \sum_h \int_{-\infty}^{\infty} dt' \; J_{r,h}(t-t') \Big(\dot{n}_h(t') - \mathbb{E}[\dot{n}_h | \{\dot{n}_r\}] \Big).$$

It has zero mean (by construction), conditioned on the activity of the recorded units — i.e., the "noise" receives feedback from the recorded neurons. We can evaluate the cross-correlation function of this noise, conditioned on the recorded unit activity. This is given by

$$\mathbb{E}[\xi_r(t)\xi_{r'}(t')|\{\dot{n}_r\}]^c = \sum_{h_1,h_2} \int_{-\infty}^{\infty} dt_1 dt_2 \; J_{r,h_1}(t-t_1) J_{r',h_2}(t'-t_2) \mathbb{E}[\dot{n}_{h_1}(t_1)\dot{n}_{h_2}(t_2)|\{\dot{n}_r\}]^c,$$

where

$$\mathbb{E}[\dot{n}_{h_1}(t_1)\dot{n}_{h_2}(t_2)|\{\dot{n}_r\}]^c = \mathbb{E}[\dot{n}_{h_1}(t_1)\dot{n}_{h_2}(t_2)|\{\dot{n}_r\}] - \mathbb{E}[\dot{n}_{h_1}(t_1)|\{\dot{n}_r\}]\mathbb{E}[\dot{n}_{h_2}(t_2)|\{\dot{n}_r\}]$$

is the cross-correlation function of the spikes (the superscript c denotes 'cumulant' or 'connected' correlation function to distinguish it from the moments without the superscript). At the level of mean field theory

$$\mathbb{E}[\dot{n}_{h_1}(t_1)\dot{n}_{h_2}(t_2)|\{\dot{n}_r\}] \approx \mathbb{E}[\dot{n}_{h_1}(t_1)|\{\dot{n}_r\}]\mathbb{E}[\dot{n}_{h_2}(t_2)|\{\dot{n}_r\}],$$

and thus the cross-correlation function is zero. We can go beyond mean field theory and calculate the tree-level contribution to the correlation functions using the field theory diagrammatic techniques of [3]. We will do so for the reference state of zero-recorded unit activity, $\{\dot{n}_r\} = \{0\}$, as we expect this to be the leading order contribution to the correlation function. As we are interested primarily in the typical magnitude of the

noise compared to the interaction terms, we will work only to leading order in $\epsilon = \exp(\mu_0)$ for the exponential nonlinearity network. We find

$$\mathbb{E}[\dot{n}_{h_1}(t_1)\dot{n}_{h_2}(t_2)|0]^c_{\text{tree}} = \int_{-\infty}^{\infty} dt' \sum_{h'} \Delta_{h_1,h'}(t_1 - t')\Delta_{h_2,h'}(t_2 - t')\nu_{h'}$$
$$\approx \lambda_0 \epsilon \delta_{h_1,h_2}\delta(t_1 - t_2),$$

where $\Delta_{h,h'}(\omega) \approx \delta_{h,h'} + \mathcal{O}(\epsilon)$ is the linear response to perturbations to the *output* of a neuron's rate. It is related to $\Gamma_{h,h'}(\omega)$ by $\Gamma_{h,h'}(\omega) = \Delta_{h,h'}(\omega)\gamma_{h'}$, where $\gamma_h = \nu_h$ for $\phi(x) = e^x$. The overall noise cross-correlation function is then approximately

$$\mathbb{E}[\xi_r(t)\xi_{r'}(t')|0]^c = \lambda_0 \epsilon \sum_h \int_{-\infty}^{\infty} dt_1 \; J_{r,h}(t - t_1)J_{r',h}(t' - t_1).$$

If $r \neq r'$, the expected noise cross-correlation, averaged over the synaptic weights $\mathcal{J}_{i,j}$, is zero. If $r = r'$, the expected value is non-zero. The expected noise auto-correlation function is then

$$\overline{\mathbb{E}[\xi_r(t)\xi_r(t')|0]^c} = \lambda_0 \epsilon N_{\text{hid}} \text{var}[\mathcal{J}] \int_{-\infty}^{\infty} dt_1 \; g(t - t_1)g(t' - t_1)$$
$$= \lambda_0 \epsilon (1 - f) J_0^2 \frac{1}{(pN)^{2a-1}} \int_{-\infty}^{\infty} dt_1 \; g(t - t_1)g(t' - t_1).$$

For the specific case of $g(t) = \alpha^2 t e^{-\alpha t}\Theta(t)$, we have

$$\overline{\mathbb{E}[\xi_r(t)\xi_r(t')|0]^c} = \frac{1}{4}\lambda_0 \epsilon (1 - f) J_0^2 \frac{1}{(pN)^{2a-1}} \alpha e^{-\alpha|t-t'|}\Big(1 + \alpha|t - t'|\Big).$$

For weak coupling ($a = 1$), the expected autocorrelation function falls off with network size as $1/N$, while for strong coupling ($a = 1/2$), it scales with the fraction of observed neurons f, but is independent of the absolute network size. The overall $\lambda_0\epsilon$ scaling puts the magnitude of the autocorrelation function on par with contributions from hidden paths through a single hidden neuron that contributes a factor of $\lambda_0\epsilon$ to the correction to the coupling filters. Based on our results shown in Fig. 5, which suggest that contributions from long paths through hidden neurons are significant when the fraction of neurons f is small and $J_0 \lesssim 1$, we expect that network noise will also be significant in these regimes. This will not modify the results presented in the main paper, however. It simply means that this noise should be retained in the rate of our approximate model,

$$\lambda_r(t) \approx \lambda_0 \exp\left(\mu_r^{\text{eff}} + \sum_{r'} J^{\text{eff}}_{r,r'} * \dot{n}_{r'}(t) + \xi_r(t)\right).$$

Validating the mean field approximation and linear conditional rate approximation via direct simulations of network activity (exponential nonlinearity)

The results presented in the main text are based on analytical calculations or numerical analyses using analytically derived formulas. For example, the statistics of $\mathcal{J}^{\text{eff}}_{r,r'}$ are calculated based on our expression $\mathcal{J}^{\text{eff}}_{r,r'} = \mathcal{J}_{r,r'} + \sum_{h,h'} \mathcal{J}_{r,h}\hat{\Gamma}_{h,h'}(0)\mathcal{J}_{h',r'}$, where $\hat{\Gamma}_{h,h'}(0)$ can be calculated by solving the matrix equation

$$\hat{\Gamma}_{h,h'}(0) = \delta_{h,h'} + \sum_{h''} \nu_h \mathcal{J}_{h,h''}\hat{\Gamma}_{h'',h'}(0).$$

Generating these results does not require a simulating the full network, so we check here that our approximations indeed agree with the results of full network simulations.

We check the validity of two main results: 1) that mean field theory is an accurate approximation for the parameters we consider, and 2) that our truncation of the conditional hidden firing rates $\mathbb{E}[\dot{n}_h(t)|\{\dot{n}_r\}]$ at linear order in $\dot{n}_r(t)$ is valid.

We first discuss some basic details of the simulation. The simulation code we use is a modification of the code used in [3], written by Gabe Ocker; refer to this paper for full details of the simulation.

The main changes we made are considering exponential nonlinearities and synaptic weights drawn from normal or lognormal distributions.

As in [3] and the main text, we choose the coupling filters to follow an alpha function

$$g_j(t) = \alpha^2 t e^{-\alpha t}\Theta(t),\ \forall j.$$

The Heaviside step function $\Theta(t)$ enforces causality of the filter, using the convention $\Theta(0) = 0$. All neurons have the same time constant $1/\alpha$.

To efficiently simulate this network the code computes the synaptic variable $s_j(t) = \int dt' g(t-t')\dot{n}_j(t')$ not by direct convolution but by solving the inhomogeneous system of differential equations, setting $x(t) = s(t)$ and $y(t) = \dot{s}(t)$,

$$\begin{aligned}\dot{x}_j(t) &= y_j(t)\\ \dot{y}_j(t) &= -2\alpha_j y_j(t) - \alpha_j^2 x_j(t) + \alpha_j^2 \dot{n}_j(t),\end{aligned}$$

The instantaneous firing rates of the neurons can in this way be quickly computed in time steps of a specified size Δt. The number of spikes n_i that neuron i fires in the tth time bin is drawn from a Poisson distribution with probability $(\lambda_i(t)\Delta t)^{n_i}\exp(-\lambda_i(t)\Delta t)/(n_i)!$. An initial transient period of spiking before the network achieves a steady state is discarded.

The parameters we use in our simulations of the full network are given in Table S1.

Table S1. Network activity simulation parameter values.

Network connectivity parameters	See Table 1.
Alpha function decay time $\tau \equiv 1/\alpha$	10
Time bin width Δt	0.01τ
Transient time window	5τ
Simulation stopping time	4000τ + transient

Validating the mean field approximation

To confirm that the mean field approximation is valid, we seek to compare the empirically measured spike rates measured from simulations of the network activity to the calculated mean field rates. The empirical rates are measured as

$$\langle \dot{n}_i \rangle^{\text{emp}} = \frac{\text{number of spikes emitted by neuron } i}{\text{length of spike train window}},$$

calculated after discarding the initial transient period of firing, for any neuron i (recorded or hidden).

The steady-state mean field firing rates are the solutions of the transcendental equation

$$\langle \dot{n}_i \rangle^{\text{full MFT}} = \lambda_0 \exp\left(\mu_i + \sum_j \mathcal{J}_{i,j} \langle \dot{n}_j \rangle^{\text{full MFT}}\right).$$

The only difference between this equation and the equation for ν_h is that the neuron indices are not restricted to hidden units. i.e., the ν_h are the mean field rates for the hidden neurons *only* (recorded neurons removed entirely), whereas the $\langle \dot{n}_i \rangle^{\text{full MFT}}$ are the mean field rates for the entire network. If the mean field approximation is valid, the empirical rates should be approximately equal to the mean field rates, so a scatter plot of $\langle \dot{n}_i \rangle^{\text{MFT}}$ versus $\langle \dot{n}_i \rangle^{\text{emp}}$ should roughly lie along the identity line. We test this for a network in the strong coupling limit ($\sqrt{\text{var}(\mathcal{J})} = J_0/\sqrt{N}$) for four values of J_0, $J_0 = 0.25, 0.5, 0.75$, and 1.0. We expect $J_0 = 1.0$ to be close to the stability threshold of the model based on a linearized analysis [4, 5]; i.e., for $J_0 \gtrsim 1.0$ there may not be a steady state, so this may be where we expect the mean field approximation to break down. As seen in Fig. S1, the mean field approximation appears to hold well even up to $J_0 = 1.0$, though there are some slight deviations for neurons with large rates.

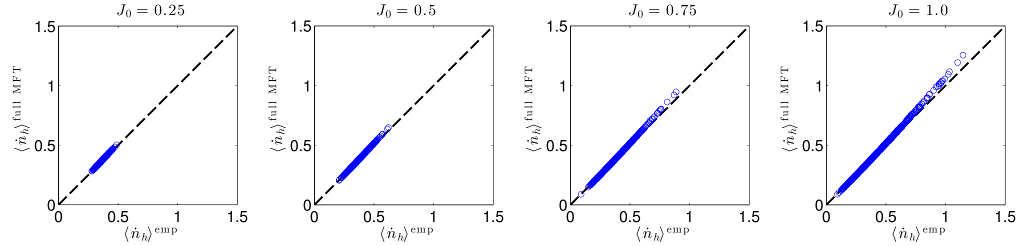

Fig S1. Empirical estimates of average neuron firing rates from simulations plotted against mean firing rates predicted by mean field theory. The fact that the data lies along the identity line demonstrates validity of the mean field theory approximation up to $J_0 = 1.0$.

Verifying the linearized conditional mean approximation

Having verified that the mean field approximation is valid, we now seek to check our linearized approximation of the firing rates of the hidden neurons *conditioned on the activity of the recorded neurons*, $\mathbb{E}\left[\dot{n}_h(t)|\,\{\dot{n}_r(t)\}\right]$. That is, we calculated above that

$$\mathbb{E}\left[\dot{n}_h(t)|\,\{\dot{n}_r(t)\}\right] \approx \nu_h + \sum_{h',r} [\Gamma_{h,h'} * J_{h',r} * \dot{n}_r](t) + \ldots;$$

the $\ldots$ correspond to higher order spike filtering terms that we have neglected in our analyses, assuming them to be small. In an earlier calculation above, we estimated that the error incurred by neglecting higher order spike filtering is of the order $(\lambda_0 \exp(\mu_0))^4 f\sqrt{1-f}J_0^3$, but we would like to confirm the negligibility of the higher order coupling through simulations.

To do so, we compare the empirical firing rates of the designated "hidden" neurons obtained from simulations of the full network models with the approximation of the firing rates of the hidden neurons conditioned on the recorded neurons using the linear expansion, averaged over recorded neuron activity to give

$$\langle \dot{n}_h \rangle^{\text{approx}} \approx \nu_h + \sum_{h',r} \hat{\Gamma}_{h,h'}(0)\mathcal{J}_{h',r}\langle \dot{n}_r \rangle^{\text{emp}},$$

where as usual the zero-frequency component of the linear response function $\hat{\Gamma}_{h,h'}(0)$ of the hidden neurons is calculated in the absence of recorded neurons.

If we make a scatter plot of this against the empirical estimates of the hidden neurons, $\langle \dot{n}_h \rangle^{\text{emp}}$, the data points will lie along the identity line if our approximation is valid. If the data deviates from the identity line, it indicates that the neglected

higher-order filtering terms contribute substantially to the firing rates of the neurons. It is possible that the zeroth order rate approximation, ν_h, would be sufficient to describe the data, so we compare the empirical rates to both ν_h and $\langle \dot{n}_h \rangle^{\text{approx}}$.

As in the mean field approximation test, we focused on a strongly coupled network with $J_0 = 0.25, 0.5, 0.75$, and 1.0. In the SI we analytically estimate the error, predicting it is small for both small and large fractions of recorded neurons and largest error when $N_{\text{rec}} \sim N_{\text{hid}}$, so we check both $N_{\text{rec}} = 100$ neurons out of $N = 1000$ neurons ($f = 0.1$) in Fig. S2 and $N_{\text{rec}} = 500$ neurons out of $N = 1000$ ($f = 0.5$) in Fig. S3.

For each value of J_0, we present two plots: the empirical rates versus the mean field rates ν_h in the absence of recorded neurons (the zeroth order approximation; Figs. S2 and S3, top row), and the empirical rates versus the linear approximation $\langle \dot{n}_h \rangle^{\text{approx}}$ (the first order approximation; Figs. S2 and S3, bottom row). We find that in both cases the data is centered around the identity line, but the spread of data grows with J_0 for the zeroth order approximation, while it is quite tight for the first order approximation up to $J_0 = 1.0$, validating our neglect of the higher order spike filtering terms. We also confirm that $N_{\text{rec}} = 500$ offers worse agreement than $N_{\text{rec}} = 100$, though the agreement between $\langle \dot{n}_h \rangle^{\text{emp}}$ and $\langle \dot{n}_h \rangle^{\text{approx}}$ is still not too bad.

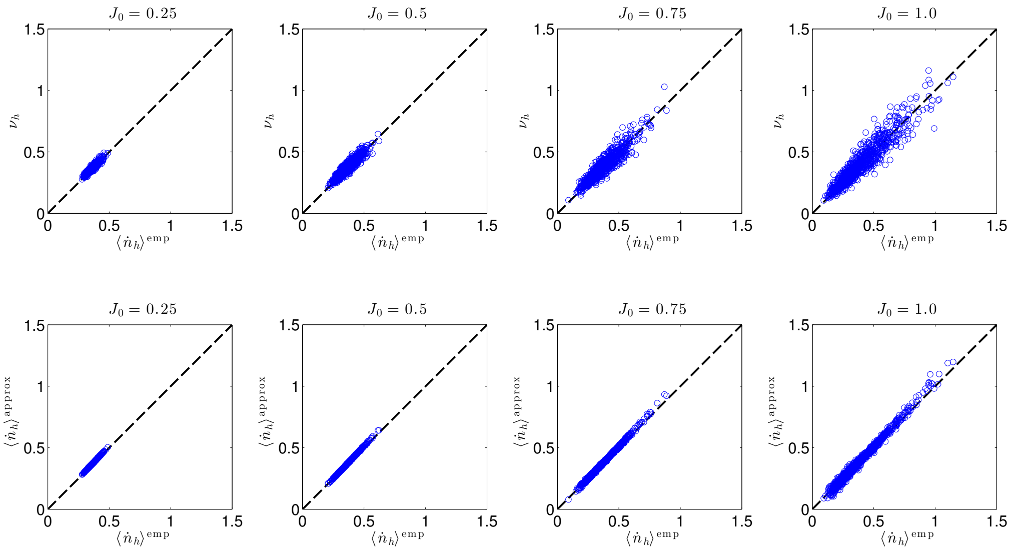

Fig S2. Top row: scatter plot comparing ν_h, the mean field firing rates of the hidden neurons in the absence of recorded neurons, to empirically estimated firing rates in simulations of the full network, for four different values of typical synaptic strength, $J_0 = 0.25, 0.5, 0.75$, and 1.0. The data lie along the identity line, demonstrating a strong correlation between ν_h and the empirical data. However, the spread of data around the identity line indicates that deviations of the mean firing rates away from ν_h, caused by coupling to the recorded neurons, is significant. **Bottom row:** Comparison of the first order approximation of the firing rates of hidden neurons, which accounts for the effects of recorded neurons, to the empirical rates. The data lie tightly along the identity with very little dispersion, demonstrating that higher order spike filtering is unnecessary even up to $J_0 = 1.0$, for $N_{\text{rec}} = 100$.

Full mean-field reference state

For most of our analyses, we have been expanding the conditional firing rates of the hidden neurons around a reference state of zero activity of the recorded neurons. The quantities ν_h, γ_h, $\hat{\Gamma}_{h,h'}(\omega)$, and so on, are thus calculated using a network in which the

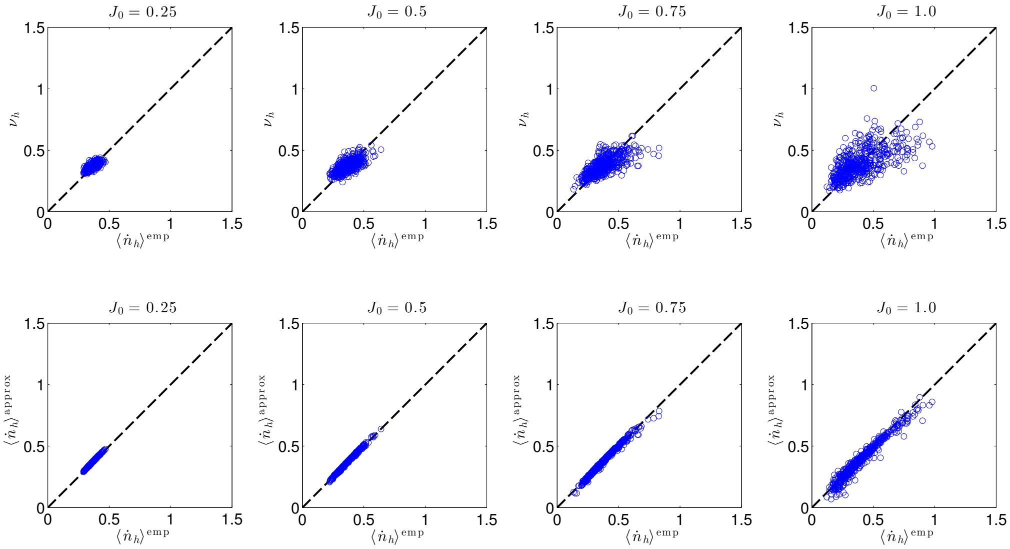

Fig S3. Same as Fig. S2 but for $N_{\rm rec} = 500$ recorded neurons out of a total of $N = 1000$. Demonstrates validity of linear approximation (neglecting higher order spike filtering) up to $J_0 = 1.0$, for $N_{\rm rec} = 500$. The zeroth order approximation (top row) is quite poor, indicating the necessity of accounting for feedback from the recorded neurons. This first order approximation (bottom row) lies tightly along the identity line, indicating that even when the recorded and hidden populations are of comparable size, higher order spike filtering may not be significant. However, there appears to be some deviation for $J_0 = 1.0$, indicating that accounting for higher order spike filtering may be beneficial in this parameter regime.

recorded neurons have been removed. We have demonstrated that this approximation is valid for the networks considered in this paper. However, this approximation may break down in networks in which the recorded neurons spike at high rates. In this case, we may need another reference state to expand the conditional rates around. A natural choice of reference state $\dot{n}_r^{(0)}(t)$ in this case would be the mean firing rates of the neurons. We will set up this expansion here.

The mean firing rates of the neurons are intractable to calculate exactly, so we will estimate them by the mean field rates, an approximation that we expect to be accurate in the high-firing rate regime.

The mean field equations for the full network are

$$\langle \dot{n}_i \rangle = \lambda_0 \phi \left(\mu_i + \sum_j J_{ij} * \langle \dot{n}_j \rangle \right).$$

We can then expand $\mathbb{E}\left[\dot{n}_h | \left\{\dot{n}_r\right\}\right]$ around $\dot{n}_r = \langle \dot{n}_r \rangle$, truncating at linear order to obtain

$$\mathbb{E}\left[\dot{n}_h(t) | \left\{\dot{n}_r\right\}\right] \approx \langle \dot{n}_h \rangle + \sum_{h',r} \int_{-\infty}^{\infty} dt' dt''\ \Gamma^{\rm full}_{h,h'}(t-t') J_{h',r}(t'-t'')(\dot{n}_r(t'') - \langle \dot{n}_r \rangle),$$

where $\Gamma^{\rm full}_{h,h'}(t-t')$ is the input linear response of the *full network*, including the recorded neurons.

We can then approximate the instantaneous firing rates of the recorded neurons by

$$\lambda_r(t) \approx \lambda_0 \phi \left(\left\{ \mu_r + \sum_{r'} J_{r,r'} * \langle \dot{n}_r \rangle \right\} + \sum_{r'} J_{r,r'} * (\dot{n}_r - \langle \dot{n}_r \rangle) + \sum_h J_{r,h} * \mathbb{E}\left[\dot{n}_h(t) \middle| \{\dot{n}_r\} \right] \right)$$
$$\approx \lambda_0 \phi \left(\left\{ \mu_r + \sum_{r'} J_{r,r'} * \langle \dot{n}_r \rangle + \sum_h J_{r,h} * \langle \dot{n}_h \rangle \right\} + \sum_{r'} \left\{ J_{r,r'} + \sum_{h,h'} J_{r,h} * \Gamma^{\text{full}}_{h,h'} * J_{h',r'} \right\} * (\dot{n}_r - \langle \dot{n}_r \rangle) \right);$$

note that we introduced $0 = \sum_{r'} \langle \dot{n}_{r'} \rangle - \sum_{r'} \langle \dot{n}_{r'} \rangle$ so that we could write the instantaneous firing not as a function of filtered spike trains but as a function of filtered deviations from the mean firing rate. Importantly, although it looks like only the baseline is different from the zero-activity reference state case but the coupling is the same, the linear response function $\Gamma^{\text{full}}_{h,h'}(\tau)$ is *not* the same as the zero-reference state case, and hence the correction to the coupling is slightly different. The solutions look similar, but the linear response functions now incorporate the effects of the recorded units as well. In particular, $\Gamma^{\text{full}}_{ij}(t - t')$ satisfies the equation

$$\int_{-\infty}^{\infty} dt'' \left[\delta_{ik} - \gamma_i^{\text{full}} J_{ik}(t - t'') \right] \Gamma^{\text{full}}_{kj}(t'' - t') = \gamma_i^{\text{full}} \delta_{ij} \delta(t - t'),$$

where γ_i^{full} is the gain of neuron i accounting for the entire network,

$$\gamma_i^{\text{full}} = \lambda_0 \phi' \left(\mu_i + \sum_j J_{ij} * \langle \dot{n}_j \rangle \right).$$

Thus, in Fourier space

$$\hat{\Gamma}^{\text{full}}_{ij}(\omega) = \left[\mathbb{I} - \hat{\mathbf{V}}^{\text{full}}(\omega) \right]^{-1}_{ij} \langle \dot{n}_j \rangle$$
$$= \sum_{\ell=0}^{\infty} \left[\hat{\mathbf{V}}^{\text{full}}(\omega))^{\ell} \right]_{ij} \langle \dot{n}_j \rangle,$$

where $\hat{V}^{\text{full}}_{i,j}(\omega) = \gamma_i^{\text{full}} \hat{J}_{i,j}(\omega)$ is an $N \times N$ matrix – i.e., it contains the couplings and firing rates of *all* neurons, recorded and hidden. Hence, while this looks formally similar to the result we obtained in the zero activity state, the inclusion of recorded neurons modifies our rules for calculating contributions to the effective coupling filters. In particular, $\hat{J}^{\text{eff}}_{r,r'}(\omega) - \hat{J}_{r,r'}(\omega)$ involves contributions from paths through both hidden and recorded neurons, unlike the zero-activity reference case, which involved contributions only from paths through hidden neurons. The reason for this, of course, is that the reference state depends on the entire network, not just the hidden neurons. The difference between the cases matters only at higher orders in our expansion — paths of length $\ell = 4$ or greater. We can see this by writing out the first few terms in the path length expansion of the effective coupling,

$$\hat{J}^{\text{eff}}_{r,r'}(\omega) = \hat{J}_{r,r'}(\omega) + \sum_h \hat{J}_{r,h}(\omega) \gamma_h^{\text{full}} \hat{J}_{h,r}(\omega) + \sum_{h,h'} \hat{J}_{r,h}(\omega) \gamma_h^{\text{full}} \hat{J}_{h,h'}(\omega) \gamma_{h'}^{\text{full}} \hat{J}_{h',r'}(\omega)$$
$$+ \sum_{h,h',j} \hat{J}_{r,h}(\omega) \gamma_h^{\text{full}} \hat{J}_{h,j}(\omega) \gamma_j^{\text{full}} \hat{J}_{j,h'}(\omega) \gamma_{h'}^{\text{full}} \hat{J}_{h',r'}(\omega) + \ldots;$$

for conciseness, we have assumed zero-self coupling ($\hat{J}_{i,i}(\omega) = 0$), but this can be restored by setting $\gamma_i^{\text{full}} \to \gamma_i^{\text{full}} / (1 - \gamma_i^{\text{full}} \hat{J}_{i,i}(\omega))$.

We see that the first few terms of the expansion are the same as the zero-activity reference case, with the exception that the γ_h^{full} are the gains for the entire network, not just the hidden network absent the recorded neurons. It is only the $\ell = 4$ term at which contributions to the linear response functions involving paths through any neuron j, recorded or hidden, start to appear. Because we typically expect the amplitude of these terms to be small, we anticipate expanding around the mean field reference state will yield similar results to the expansion around the zero-activity reference state presented in the main paper.

References

1. Gerstner W, Kistler WM, Naud R, Paninski L. Neuronal Dynamics: From single neurons to networks and models of cognition. Cambridge, U.K.: Cambridge University Press; 2014.
2. Latimer K, Chichilnisky E, Rieke F, Pillow J. In: Inferring synaptic conductances from spike trains under a biophysically inspired point process model. vol. 2. january ed. Neural information processing systems foundation; 2014. p. 954–962.
3. Ocker GK, Josić K, Shea-Brown E, Buice MA. Linking structure and activity in nonlinear spiking networks. PLOS Computational Biology. 2017;13(6):1–46. doi:10.1371/journal.pcbi.1005583.
4. HAWKES AG. Spectra of some self-exciting and mutually exciting point processes. Biometrika. 1971;58(1):83. doi:10.1093/biomet/58.1.83.
5. Brémaud P, Massoulié L. Stability of nonlinear Hawkes processes. Ann Probab. 1996;24(3):1563–1588. doi:10.1214/aop/1065725193.
